# Development of novel multi-protein chimeric immunogens that protect against infection with the Lyme disease agent, *Borreliella burgdorferi*

**DOI:** 10.1128/mbio.02159-24

**Published:** 2024-09-17

**Authors:** Nathaniel S. O’Bier, Andrew C. Camire, Dhara T. Patel, John S. Billingsley, Kelly R. Hodges, Richard T. Marconi

**Affiliations:** 1 Department of Microbiology and Immunology, Virginia Commonwealth University, Richmond, Virginia, USA; University of California, Irvine, California, USA

**Keywords:** *Borreliella*, *Borrelia*, OspA, OspB, FtlA, FtlB, chimeritope, chimeric, Lyme disease vaccine

## Abstract

Lyme disease is the most common tick-borne disease in North America. A vaccine for use in humans is not available. Here, we detail the development of two chimeric vaccine antigens, BAF and Chv2M. BAF elicits Abs that target proteins and protein variants produced by *Borreliella* species in ticks (OspB and OspA) and mammals (FtlA/B). OspB serves as the backbone structure for the BAF chimeric. Two OspA_221-240_ epitope-containing domain (ECD) variants (#A1 and #A15) were inserted into a loop in OspB. The N-terminal region of the FtlA protein was joined to the C-terminus of the chimeric. The second chimeric, Chv2M, consists of L5 (loop 5) and H5 (helix 5) ECDs derived from diverse OspC proteins. *Borreliella* species produce OspC upon exposure to the bloodmeal and during early infection in mammals. Here, we demonstrate that BAF and Chv2M are potent immunogens that elicit Abs that bind to each component protein (FtlA, FtlB, OspB, and multiple OspA and OspC variants). Anti-BAF and anti-Chv2M Abs kill *Borreliella burgdorferi* strains through Ab-mediated complement-dependent and complement-independent mechanisms. Eighty percent (32/40) of mice that received a three-dose vaccine regimen were protected from infection with *B. burgdorferi* B31. Efficacy increased to 90% (18/20) when the amount of Chv2M was increased in the third vaccine dose. Readouts for infection were *flaB* PCR and seroconversion to VlsE. This study establishes proof of principle for a chimeric immunogen vaccine formulation that elicits Abs to multiple targets on the *B. burgdorferi* cell surface produced during tick and mammalian stages of the enzootic cycle.

**IMPORTANCE:**

Lyme disease is a growing public health threat across parts of the Northern Hemisphere. Regions that can support sustained tick populations are expanding, and the incidence of tick-borne diseases is increasing. In light of the increasing risk of Lyme disease, effective preventive strategies are needed. Most vaccine development efforts have focused on outer surface protein A, a *Borreliella burgdorferi* protein produced only in ticks. Herein, we describe the development of a novel vaccine formulation consisting of two multivalent chimeric proteins that are immunogenic and elicit antibodies with bactericidal activity that target several cell surface proteins produced by the Lyme disease spirochetes in feeding ticks and mammals. In a broader sense, this study advances efforts to develop custom-designed vaccinogens comprised of epitope-containing domains from multiple proteins.

## INTRODUCTION

Lyme disease (LD) is the most common tick-borne disease in the Northern Hemisphere (1, 2). It is estimated that more than 470,000 and 200,000 new human LD cases occur each year in the USA (1) and Europe (3), respectively. The primary causative agent of LD in North America is *Borreliella burgdorferi* (1). Additional *Borreliella* species, including *Borreliella garinii, Borreliella afzelii,* and *Borreliella bavariensis*, are associated with human disease in Europe and Asia (3).

Ongoing industry efforts to develop a human LD vaccine have centered on OspA (4, 5), a lipoprotein produced during the tick phase of the enzootic cycle of the LD spirochetes. The utility of OspA as a vaccine antigen was demonstrated decades ago (6). The OspA-based vaccine, LymeRix, entered the human vaccine market in 1998 but was discontinued in 2002 (7). The mechanism of action of LymeRix was to elicit Abs that, when taken up by feeding ticks, can target OspA on the surface of *B. burgdorferi* in the tick midgut and prevent transmission. While transmission-blocking vaccines are conceptually attractive, there are potential shortcomings. Protection is dependent on the maintenance of high-circulating Ab titers. In addition, since OspA is not produced in mammals, vaccinated individuals do not mount an OspA-directed memory immune response upon exposure to infected ticks; thus, frequent booster immunizations are required to maintain protection.

LymeRix consisted of a single variant of lipidated OspA (variant 1). While OspA is a well-conserved protein among *Borreliella* species in N. America, six additional phyletic types (also referred to as serotypes) have been identified in Europe. Valneva developed a multi-valent OspA vaccine formulation (VLA15) consisting of three lipidated OspA heterodimers, each comprised of the C-terminal domain of two different OspA variants (8–10). The primary intent of the multivalent OspA approach is to develop a vaccine formulation that will protect against strains circulating in Europe and N. America.

We have pursued the development of a multi-valent, multi-target LD vaccine formulation using chimeric immunogens. An inherent advantage of recombinant chimeric immunogens is that they can be constructed to contain protective epitopes from multiple proteins or protein variants [reviewed in reference (11)]. In addition, regions of a protein that harbor non-protective epitopes can be excluded, resulting in focused and productive Ab responses. Chimeric epitope-based vaccines are being pursued for several important infectious diseases/pathogens, including influenza, monkeypox, *Chlamydia*, *Leishmania*, and SARS-CoV-2 (12–18). Previously, we developed a novel recombinant subunit Lyme disease vaccine for dogs called Vanguard crLyme (19). To our knowledge, crLyme was the first chimeric epitope-based vaccine approved by the USDA that has been successfully commercialized. The two-protein subunit vaccine formulation consists of non-lipidated OspA (variant 1; also referred to as serotype 1) and a laboratory-designed OspC-based chimeric epitope (chimeritope) protein (CH14) (19). CH14 comprises an OspC type F backbone structure, joined with 14 contiguous OspC L5 and H5 epitope-containing domains (ECDs) from diverse *B. burgdorferi* strains (20). This unique OspC chimeritope was designed to elicit Abs against diverse OspC proteins. Immunization of purpose-bred beagles with two doses of crLyme (3 weeks apart) prevented seroconversion and infection when challenged with field-collected *Ixodes scapularis* ticks. A field safety study that included 620 dogs demonstrated that the vaccine was safe and elicited infrequent mild adverse events (21).

Here, we detail the development of a new LD chimeric epitope subunit vaccine formulation. The vaccine is comprised of two chimeric proteins (BAF and Chv2M). BAF consists of ECDs from OspB, OspA (22), and FtlA/FtlB (23). We previously demonstrated that the OspA_221-240_ ECD, when conjugated to KLH, elicits Abs in mice with bactericidal activity nearly equivalent to full-length OspA (22). The N-terminal domain of FtlA, a protein produced over long-term infection in mammals, also harbors bactericidal epitopes (23). The Chv2M chimeric comprises a series of 20 L5 and H5 ECDs derived from diverse OspC types (20, 24). Immunization of rats and mice immunized with the chimerics demonstrated that the proteins are immunogenic and elicit Abs that surface-label all cells in a *B. burgdorferi* population and have broad bactericidal activity. Challenge studies demonstrated that 80% (32 of 40) of mice that received three doses of BAF/Chv2M were protected from infection with *B. burgdorferi*. Increasing the amount of Chv2M in the third vaccine dose increased protective efficacy to 90% (18/20). The results support the advancement of a novel two-chimeric protein vaccine formulation for preventing LD.

## MATERIALS AND METHODS

### Bacterial strains and cultivation

*Borreliella* strains (described in Table 1) were cultivated in BSK-H complete media supplemented with 6% rabbit serum (Sigma) at 34°C. Growth was monitored using wet mounts and dark-field microscopy.

**TABLE 1:** *Borreliella* strains and mutants used in this study

Species/strain	Biological origin, literature reference, or provider	Description
*B. burgdorferi* B31 (clone 5A4)	*Ixodes scapularis* tick, New York, USA (25)	Clonal derivative of the B31 isolate. OspA variant 1; OspC type A
*B. burgdorferi* B31OspC KO	As above	*ospC* knock out (KO) (26). OspA variant 1
*B. burgdorferi* B31 OspA KO and OspB KO	As above	*ospA* and ospB KO mutants (27); OspA variant 1; OspC type A
*B. burgdorferi* B31-HE-OspC	As above	Constitutive expression of OspC (28). OspA variant 1; OspC type A
*B. burgdorferi* LDP60	Human blood, Maryland, USA (20)	OspA variant 1; OspC type A
*B. burgdorferi* LDP74	Human blood, Maryland, USA (20)	OspA variant 1; OspC type K
*B. burgdorferi* LDP73	Human blood, Maryland, USA (20)	OspA variant 1; OspC type B
*B. burgdorferi* CA2-87	*Ixodes pacificus* tick, California, USA (29)	OspA variant 1; OspC type A
*B. burgdorferi* CA12	*Ixodes pacificus* tick, California, USA; Dr. T.G. Schwan	OspA variant 1; OspC type M
*B. burgdorferi* IP2A	Human cerebrospinal fluid, France (30)	Not determined
*B. burgdorferi* VS134	*Ixodes ricinus* tick, Switzerland; Dr. T.G. Schwan	OspA variant 1; OspC type L
*B. afzelii* ECM1	Human erythema migrans biopsy, Sweden; Dr. T.G. Schwan	OspA variant 2; OspC type A2

### Cloning and gene synthesis

**Fig 1:**
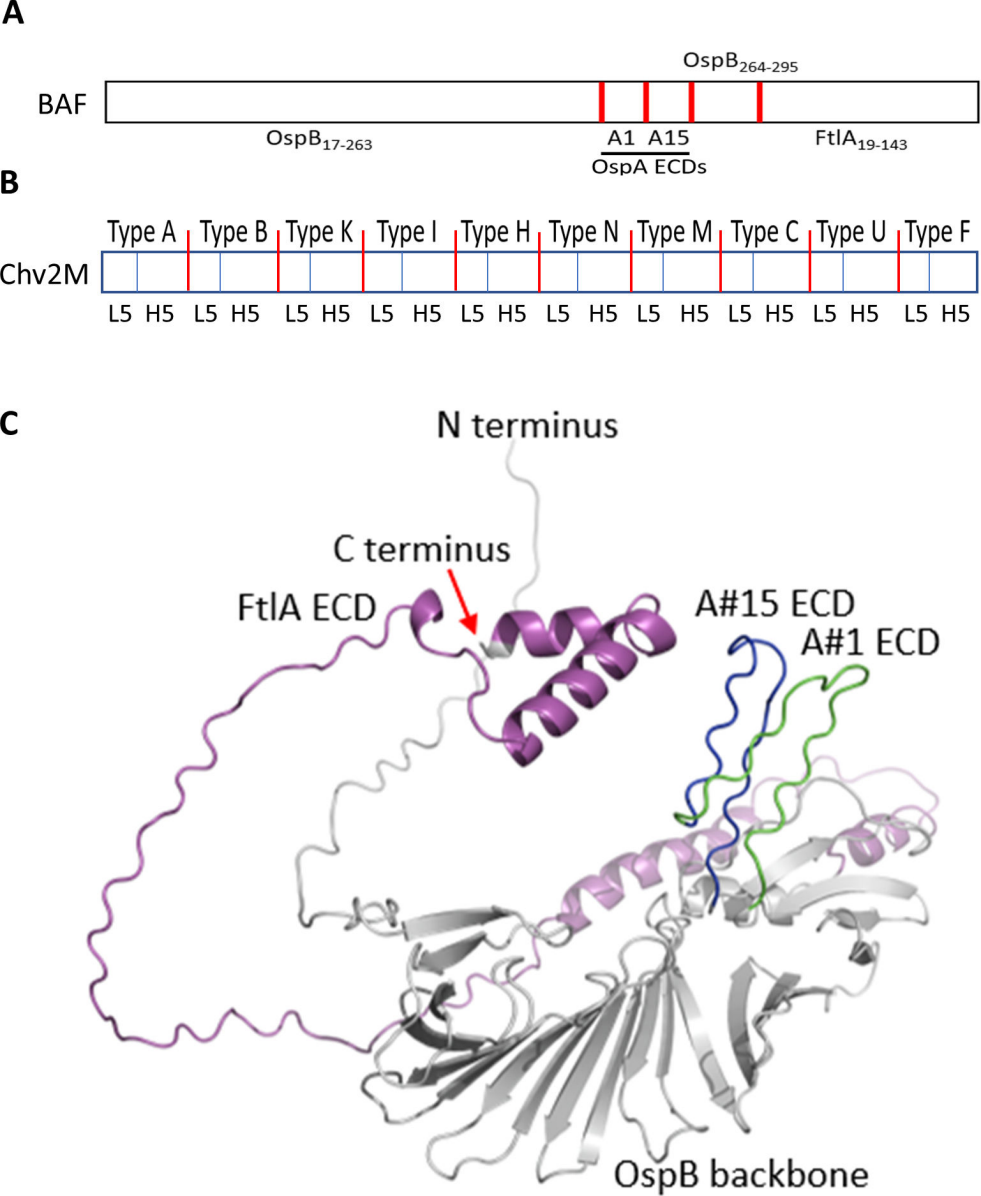
Structural predictions of BAF and schematic representations of the chimeric organization of the BAF and Chv2M immunogens. Panel (A) presents a linear schematic representation of the organization of BAF with the ECDs indicated. The boundaries of each chimeric segment are indicated with red vertical bars. Panel (B) depicts the ECD organization of Chv2M. The OspC types represented in the protein are bracketed by red vertical lines and labeled along the top. The segments corresponding to the loop 5 (L5) and helix 5 (H5) ECDs are indicated with blue vertical lines. The predicted structure of BAF (panel C) was generated as detailed in the text. The individual domains of the chimeric are indicated. FltA is highlighted in magenta, OspB in gray, OspA_221-240_ #A1 ECD in green, and OspA #A15 ECD in blue.

Genes encoding recombinant proteins (OspA variants 1, 2, 3, 4, 5, and 6; OspB, FtlA, FtlB, and VlsE; and OspC types A, A2, B, C, D, F, G, H, I, J, K, L, M, N, T, U, Jem5, SZ94, Phi, Phoe, B5/92, Pwa, and *Borrelia mayonii*) and the chimeric antigens (Chv2M and BAF) were codon-optimized, synthesized, and inserted into the multi-cloning site of pET45b(+) at the BamHI and EagI restriction sites (fee-for-service; Genscript). Leader-encoding sequences were omitted from all synthesized genes to facilitate expression in *Escherichia coli* BL21(DE3) cells. The BAF chimeric consists of an OspB backbone, two OspA_221-240_ ECD variants (#A1 and #A15) (22), and the N-terminal domain of FtlA (Fig. 1A). Sequences that encode the OspA_221-240_ #A1 and #A15 ECDs (22) were incorporated into the construct between the codons for OspB amino acid residues, Asp_264_ and Gly_265_, to form the *ospB-A1-A15* construct. The selection of this site for the insertion of the OspA_221-240_ ECDs was based in part on the determined structure of a segment of OspB spanning residues Ser_157_ to Lys_296_, which indicates that Asp_264_ and Gly_265_ are located within a short surface-exposed loop (31). The sequence encoding the N-terminal immunodominant domain of *B. burgdorferi* B31 FtlA (residues 19–143) (23) , preceded by two added Trp codons included to increase the extinction coefficient and detection sensitivity of the protein, was added to the 3′-end of the *ospB-A1-A15* construct to yield *BAF*. The FtlA_19-143_ domain is disordered, flexible, and surface-exposed (32) and harbors epitopes that elicit bactericidal Abs (23). The resulting hexa-histidine-tagged BAF protein has a predicted molecular weight (MW) of 52.2 kDa (472 amino acids) and an isoelectric point of 6.76. The gene encoding Chv2M comprises a series of 20 OspC L5 and H5 ECDs derived from diverse OspC types (Fig. 1B). Note that some L5 and H5 ECDs were truncated to minimize repetitive sequences. The *chv2M* gene encodes a chimeric protein with a predicted MW of 54.7 kDa (486 amino acids) and an isoelectric point of 6.01. Protein properties were determined using Protparam (33). Alphafold (34) was used to model the structure of BAF. Molecular graphics and analyses were performed with ChimeraX (35). The output pdb file was then exported into PyMOL for final visualization and editing.

### Protein production and purification

Recombinant plasmids were propagated in *E. coli* NovaBlue cells, purified using Qiagen Mini-prep spin columns, and transformed into *E. coli* BL21(DE3) cells, and recombinant protein production was induced with 1.0-mM isopropyl-β-D-thiogalactopyranoside (32). The cells were harvested by centrifugation and lysed using a high-pressure cell homogenizer (Avestin). Recombinant proteins that partitioned with the *E. coli* soluble phase (VlsE, FtlA, FtlB, all OspC types, all OspA variants, OspB, Chv2M, and BAF) were purified via their N-terminal hexa-his tags using nickel affinity chromatography on an ÄKTA Fast Protein Liquid Chromatography purification platform (Cytiva) or by gravity flow through His-Bind resin columns (36). As previously described, size exclusion chromatography and SDS-PAGE were used to assess the oligomeric state, aggregation, purity, and protein integrity (37). To ensure that Abs raised against the his-tagged chimeric immunogens do not react with the his-tag of VlsE, the his-tag was cleaved from VlsE using the light chain of enterokinase (NEB). Recombinant VlsE (250 µg) was incubated with enterokinase (160 units) in cleavage buffer (20 mM Tris-HCl, 50 mM NaCl, 2 mM CaCl_2_; pH 8.0; 16 hr; 25°C), and then, the enterokinase was removed by affinity chromatography with trypsin inhibitor agarose as instructed by the supplier (Sigma-Aldrich). The flowthrough was collected and dialyzed into phosphate-buffered saline (PBS; pH 7.4). Cleavage and removal of the his-tag were confirmed by SDS-PAGE and immunoblot analysis using anti-his Ab (Invitrogen) and anti-VlsE antiserum.

### SDS-PAGE and immunoblot analyses

Recombinant proteins (500 ng) were fractionated by SDS-PAGE in Criterion AnykD Gels (Bio-Rad) and transferred to polyvinylidene fluoride membranes (0.22 µm pore size) using the Trans-Blot Turbo system as per the manufacturer instructions (Bio-Rad). The membranes were incubated with PBS-MT blocking solution (PBS with 5% non-fat dried milk and 0.2% Tween-20; 1 hr; room temperature), followed by the addition of antiserum or infection serum from mice (1:1,000 in PBS-MT). The sera were decanted, the blots were washed three times with PBS-T (PBS with 0.2% Tween-20), and horseradish peroxidase (HRP)-conjugated secondary Abs were added in PBS-MT (1:40,000 dilution). Bound IgG was detected by adding the Clarity Western ECL substrate (5 min; room temperature; Bio-Rad). Images were captured using a ChemiDoc Touch imaging system (Bio-Rad; auto-optimal settings) and cropped for presentation.

### Generation of antisera

Antiserum to recombinant proteins was generated in Sprague–Dawley rats (females; body weight approximately 165 g; Charles River), as previously described (22). In brief, 50 µg of recombinant protein in Freund’s Complete adjuvant (Sigma) was injected into the peritoneal cavity. Boosts of 25 µg of each protein in Freund’s Incomplete adjuvant (Sigma) were delivered 2 and 4 weeks later. One week after the last immunization, the rats were euthanized by CO_2_ asphyxiation, and blood was collected by cardiac puncture. Serum was harvested using Z Serum Sep Clot Activator columns as instructed by the supplier (Thermo Fisher). All animal experiments were conducted following the *Guide for the Care and Use of Laboratory Animals* (Eighth edition) and in accordance with protocols peer-reviewed and approved by the Virginia Commonwealth University Institutional Animal Care and Use Committees (protocol #: AD10000387).

### Differential scanning fluorimetry

The melting temperature (Tm) of the recombinant proteins was determined using differential scanning fluorimetry thermal shift assays. The recombinant proteins were diluted to 1 mg mL^−1^ in H_2_0. A 50× stock solution of SYPRO Orange was made by dilution from a 5,000× stock (Invitrogen). Ten micrograms of protein (1 mg mL^−1^) was aliquoted into the wells of a 384-well plate (Bio-Rad). The SYPRO Orange (2.5 µL) and 1× PBS pH 7.4 (12.5 µL) were added to each well for a total volume of 25 µL per well. The plates were sealed, run under a melt curve range of 10.0°C–95.0°C (increments of 0.5°C per 10 s), and read using the FRET channel in a CFX Opus 384 Real-Time PCR system (Bio-Rad). All samples were tested in triplicate.

### Indirect enzyme-linked immunosorbent assays

Enzyme-linked immunosorbent assays (ELISAs) were conducted using 96-well plates (Thermo Fisher) coated with 500 ng of recombinant protein (1 hr; 37°C; bicarbonate buffer). The ELISA plate wells were washed six times with PBST using an automated plate washer (BioTek). Serum samples (diluted 1:1,000 in PBS-MT) were added. The plates were incubated (1 hr; room temperature) and washed as above. HRP-conjugated goat anti-mouse IgG was added (diluted 1:15,000 in PBS-MT), and the plates were incubated (1 hr; room temperature). After washing, the 2,2′-azino-bis-(3-ethylbenzothiazoline-6-sulfonic acid) (ABTS) substrate was added, and the plates were incubated in the dark for 20 min. Absorbance at 405 nm was measured using a BioTek ELx808 plate reader (BioTek). Preimmune (PI) serum and bovine serum albumin (BSA) served as negative controls. All samples were tested in triplicate.

### Indirect immunofluorescence assays

Immunofluorescence assays (IFAs) were performed as previously described (22). *B. burgdorferi* cells (midlog phase) were spotted on Superfrost Plus slides (Fisher Scientific) and air-dried (30 min). The slides were blocked with PBS-BT (PBS with 3% BSA; 0.2% Tween-20), rinsed, and overlaid with the desired antiserum (diluted 1:500 in PBS-BT; 30 min; room temperature). The slides were rinsed and overlaid with Alexa-Fluor 568 goat anti-rat IgG in PBS-BT (1:500; 30 min; in the dark; Molecular Probes). The slides were washed again, ProLong Gold Antifade Reagent with DAPI (Molecular Probes) was applied, and coverslips were mounted. IgG binding was detected by fluorescence microscopy using a BX53 microscope equipped with a DP74 camera (Olympus) at 400× magnification. Images of the cells in the same fields of view were obtained using dark-field microscopy.

### Bactericidal assays

Sera from rats immunized with BAF, Chv2M, and BAF and Chv2M in combination, as detailed above, were assessed for bactericidal activity using *B. burgdorferi* B31-HE-OspC cells as the test strain as previously described (36). Guinea pig serum (GPS; Complement Tech) served as the exogenous complement source. All assays were performed in triplicate. Rat-anti-OspA (variant 1) antiserum served as the positive control for bactericidal activity. The total number of live cells in five representative fields of view was counted and averaged. Percent killing was calculated by comparing the number of live cells counted in treatment groups with the number of live cells incubated with PI serum and GPS. Statistical significance was assessed using one-way ANOVA with Tukey’s multiple comparisons test (95% CI, *P* < 0.0001), comparing all conditions to the PI control. All calculations were performed using GraphPad Prism 9.2.0 (GraphPad).

The bactericidal activity of the rat anti-BAF/Chv2M was tested against *B. burgdorferi* strains and *ospA, ospB,* and *ospC* deletion mutants, as detailed above, with important modifications. A previous study demonstrated that *B. burgdorferi* strains differ significantly in their sensitivity to GPS (38). Hence, before conducting the bactericidal assays, we assessed the sensitivity of each strain using a new lot of GPS (Lot #10; manufacturer-determined functional activity of 1,450 units/mL; Complement Tech). Several of the test strains were killed by 20% GPS alone. Hence, to determine the appropriate concentration of GPS to use in these assays, the test strains (Table 1) were incubated in 0%–20% GPS. The GPS concentration that did not in and of itself result in Ab-independent complement-mediated killing was determined to be 2.5%. In brief, 4 µL of each culture was incubated with 20% heat-inactivated (HI)-anti-BAF/Chv2M antiserum and 2.5% GPS. The volume was brought to 20 µL with BSK-H media, and the samples were incubated overnight at 37°C. The assays were also conducted with HI-GPS to determine if killing occurs through an Ab-mediated complement-independent mechanism. Cells from the treatment and control groups were counted, and the percent killing was determined as detailed above.

### Vaccination and challenge studies

C3H/HeN mice (6–8 weeks of age; equal numbers of males and females; Jackson Labs) were divided into eight study groups (Table 2). Three doses of the BAF/Chv2M formulation (Imject alum; Thermo Fisher) were administered to study groups 1, 2, 3, 5, 6, and 7. For groups 3 and 7, the amount of Chv2M delivered in the third vaccine dose was increased from 25 to 50 µg. Control groups 4 and 8 received adjuvant alone. On day 42, mice in groups 2–4 and 6–8 were challenged with *B. burgdorferi* B31 (10^4^ cells) delivered by subcutaneous needle injection between the shoulder blades. All mice were euthanized on day 63 by CO_2_ asphyxiation. Ear tissue (4 × 4 mm) and blood (cardiac puncture) were collected, and sera were harvested.

**TABLE 2:** Mouse study groups, treatment, and challenge results (summary)

Group #	Animal ID #s	Sex	Vaccine dose/timeline	*B. burgdorferi* challenge (day 42)	Protected from infection (VlsE Ab and *flaB* PCR negative). Day 63
1	161–170	F	25 µg BAF/25 µg Chv2 Days 0, 14, and 28	No: mice were sacrificed, and serum was harvested	Not applicable
2	171–180	F	25 µg BAF/25 µg Chv2M Days 0, 14, and 28	Yes	8/10
3	181–190	F	25 µg BAF/25 µg Chv2M Days 0 and 14; 25 µg BAF/50 µg Chv2M on day 28	Yes	9/10
4	191–200	F	Adjuvant only. Days 0, 14, and 28	Yes	0/10
5	201–210	M	25 µg BAF/25 µg Chv2M Days 0, 14, and 28	No: mice were sacrificed, and serum was harvested	Not applicable
6	211–220	M	25 µg BAF/25 µg Chv2M Days 0, 14, and 28	Yes	6/10
7	221–230	M	25 µg BAF/25 µg Chv2M Days 0 and 14; 25 µg BAF/50 µg Chv2M on day 28	Yes	9/10
8	231–240	M	Adjuvant only. Days 0, 14, and 28	Yes	0/10

### Ab isotyping

ELISA plate wells were coated with recombinant proteins (500 ng per well, as above). Sera from immunized mice were added (1:2,500 dilution), and the plates were incubated for 1 hr at room temperature. After washing, isotype-specific monoclonal Abs were added (Thermo Fisher; 1:2,000 dilution in blocking buffer). The plates were washed six times with PBST (automated washer), the ABTS substrate was added (20 min; room temperature), and absorbance at 405 nm was measured using a BioTek ELx808 plate reader.

### DNA isolation from tissue and PCR

Tissue biopsies (ear; approximately 4 × 4-mm sections) were collected from mice in study groups 3, 4, 7, and 8, and DNA was extracted using the DNeasy Blood and Tissue Kit (Qiagen) as per the manufacturer’s protocol. PCR was conducted using Phusion polymerase (Thermo) and primers specific for the *B. burgdorferi flaB* gene (FWD: 5′-CAGGTAACGGCACATATTCAGATGC-3′; REV: 5′-CTTGGTTTGCTCCAACATGAACTC-3). After an initial denaturation step (98°C; 30 s), 35 cycles of denaturation (98°C; 20 s), annealing (61°C; 30 s), and extension (72°C; 30 s) with a final extension step of 72°C for 7 min were conducted. The positive control reaction used DNA extracted from the tissue of a mouse previously confirmed to be infected with *B. burgdorferi* B31 as an amplification template.

## RESULTS

### Chimeric antigen production, purification, protein properties, and immunogenicity

**Fig 2:**
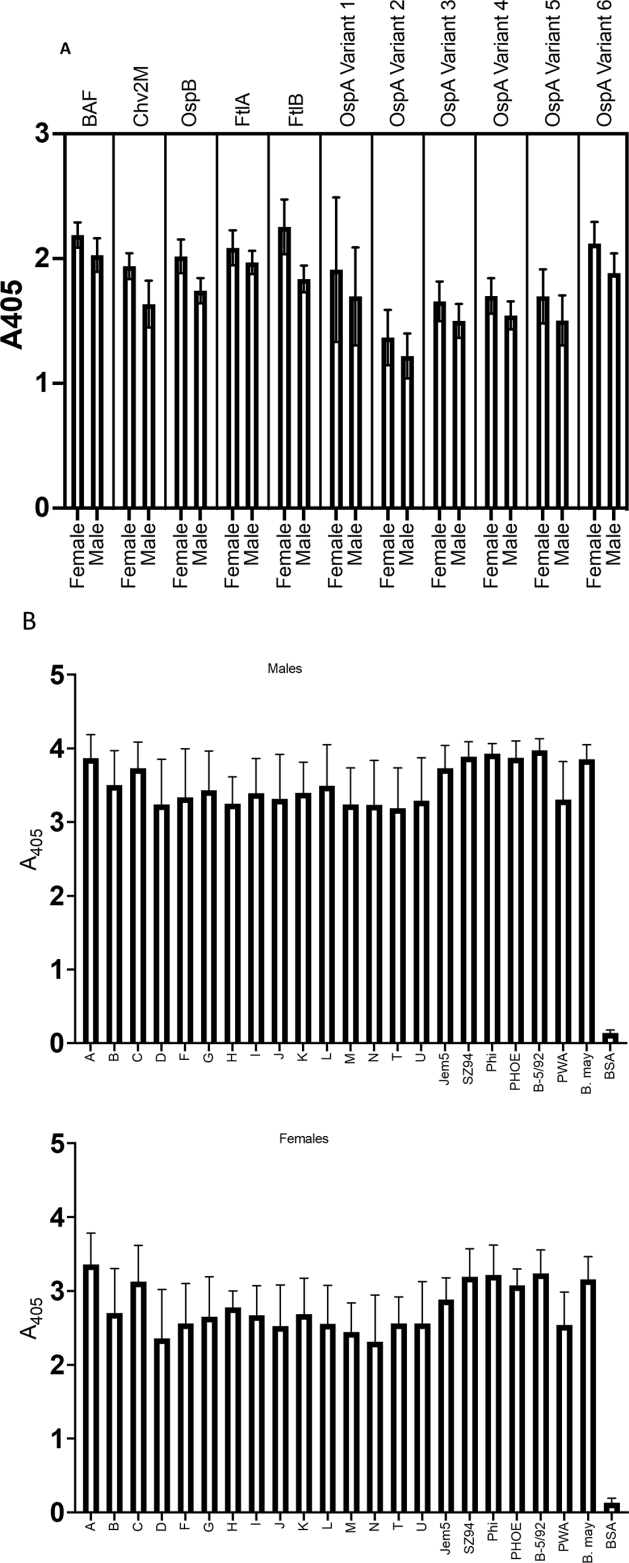
Ab responses to BAF, Chv2M, OspB, FtlA, FtlB, OspA (variants 1–6), and diverse OspC-type proteins. Individual sera from female and male mice (as indicated on the *x*-axis) immunized with BAF/Chv2M (groups 1 and 5; see Table 2) were screened by ELISA for Abs to the proteins listed above each bar graph. In panel (B), diverse OspC types from North American and European strains of the Lyme disease spirochetes were screened with pooled serum from group 1 male (top panel) and group 5 female (bottom panel) mice (see Table 2). The OspC-type designations are indicated along the *x*-axis. All methods are detailed in the text. BSA served as a negative control.

The BAF and Chv2M chimeric proteins were readily expressed in *E. coli,* and both partitioned with the soluble phase. The approximate yield of each purified protein was approximately 45 mg/L of induced culture. The organization of the chimeric proteins is shown in schematic form in Fig. 1A and B. The structure of BAF was modeled using Alphafold (Fig. 1C). To determine if the introduction of ECDs into OspB adversely affects thermal stability, the Tm of OspB and BAF were determined and compared using differential scanning fluorimetry thermal shift assays. The Tm of OspB and BAF in PBS were 53.5°C and 54°C, respectively, indicating that the organization of the chimeric protein does not significantly impact thermal stability (data not shown). ELISA analyses (single-point dilution) of serum from rats immunized with BAF and Chv2M alone or in combination demonstrated the immunogenicity of chimerics (data not shown). To verify immunogenicity in mice, sera from mice in groups 1 and 5 (Table 2) were separately pooled and screened by ELISA (1:1,000 dilution) for Abs to BAF, Chv2M, FtlA, FtlB, 6 OspA variants (Fig. 2A), and 23 OspC proteins (Fig. 2B and C). All recombinant proteins were immunoreactive with the mouse anti-BAF/Chv2M antisera. It is noteworthy that immunization with BAF and Chv2M elicited Abs that bound to all major variants of OspA and OspC tested including several European OspC types and the OspC protein of the recently identified N. American species, *B. mayonii* (Fig. 2B) (39, 40).

### Anti-BAF/Chv2M Abs label the cell surface of all cells in a *B. burgdorferi* B31 population

**Fig 3:**
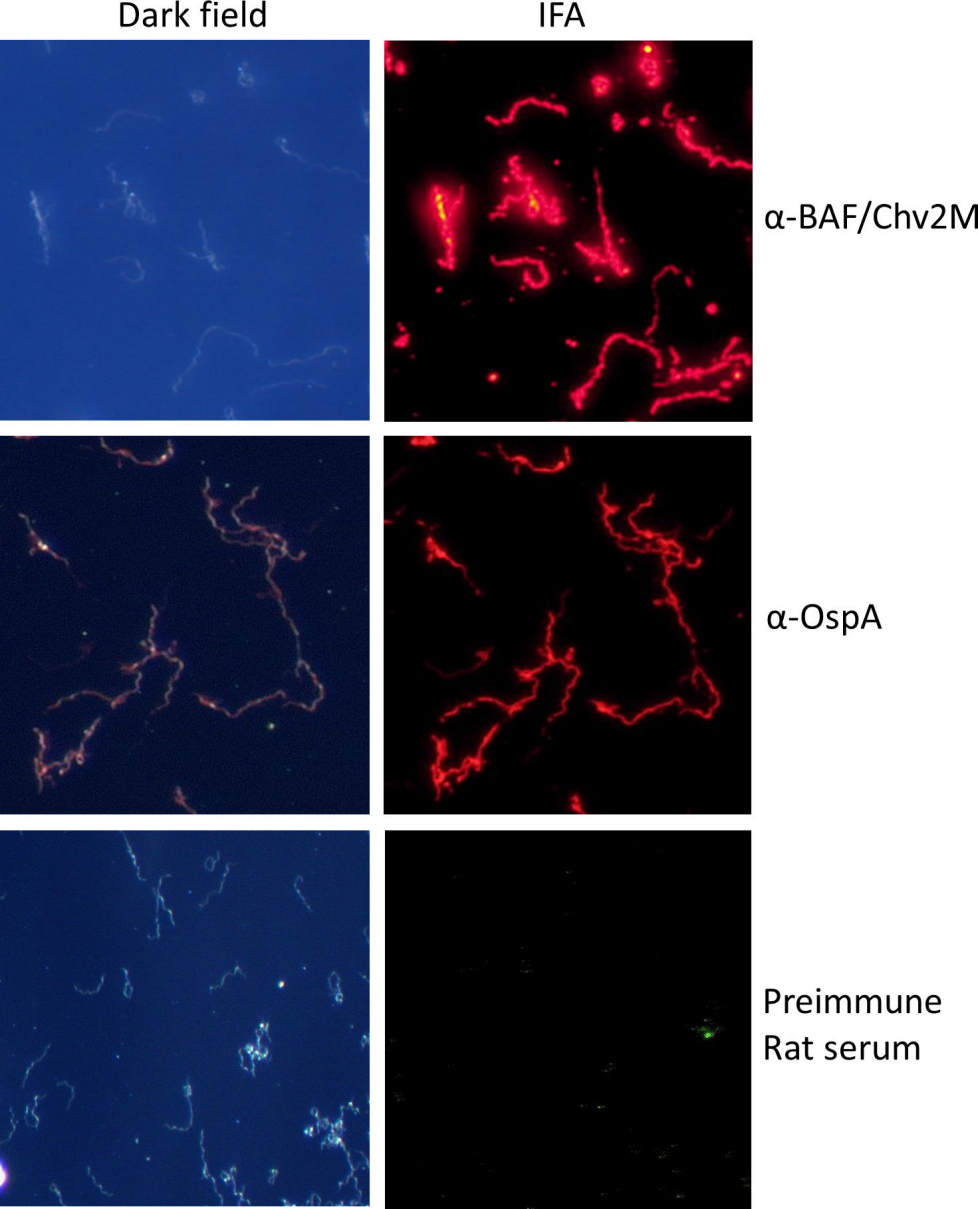
Anti-BAF/Chv2M Abs bind to all *B. burgdorferi* B31 cells. Actively growing cultures of *B. burgdorferi* B31 strains were spotted on slides and air-dried for IFA analyses, as detailed in the text. Dark-field (DF) and IFA images are provided. The slides were screened with the rat antisera or preimmune rat serum as indicated to the right. Anti-OspA antiserum served as a positive control.

To determine if anti-BAF/Chv2M Abs bind to the surface of all cells in a population, IFAs were performed using air-dried non-permeabilized cells. Anti-BAF/Chv2M serum from mice and anti-OspA antiserum (positive control) labeled the surface of all *B. burgdorferi* B31 cells (Fig. 3). Fluorescence was not observed with cells incubated with preimmune serum. The IFA analyses demonstrate that all cells in the population are presenting OspA, OspC, or FtlA domains on the surface suggesting that all cells can be targeted by vaccinal Ab.

### Abs to BAF and Chv2M are bactericidal and kill through Ab-mediated complement-dependent and complement-independent mechanisms

**Fig 4:**
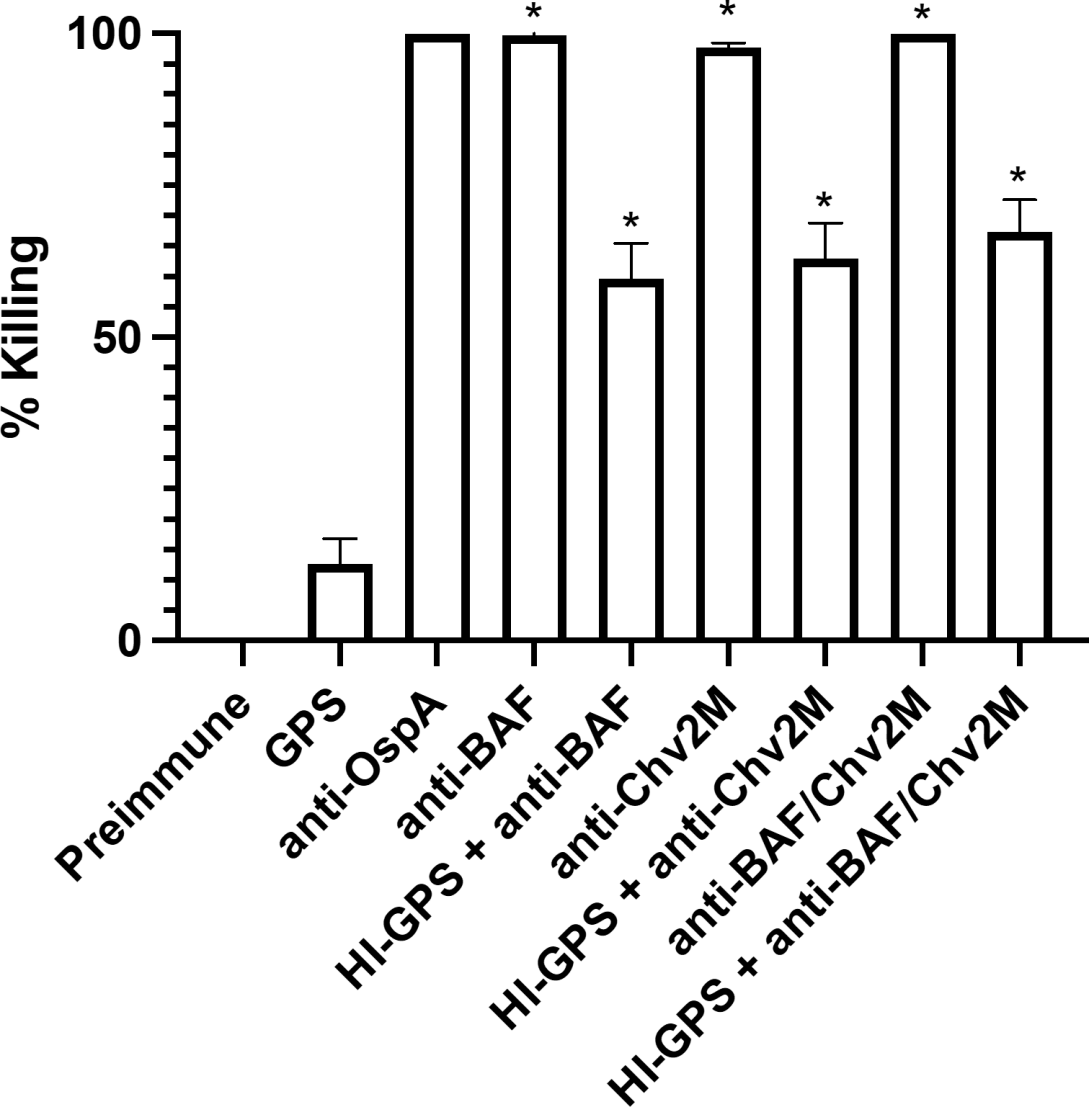
Demonstration that BAF and Chv2M elicit bactericidal Abs that kill through Ab-mediated complement-dependent and complement-independent mechanisms. The bactericidal activity of Abs raised against BAF, Chv2M, and BAF/Chv2M in rats was assessed using an *in vitro* correlate of protection assay detailed in the text. *B. burgdorferi* B31-HE-OspC (constitutive OspC expression) served as the test strain. The HI antisera were incubated with cells with GPS or HI-GPS. Additional controls included cells incubated with anti-OspA antiserum (with GPS; positive control for bactericidal activity) and cells incubated with media supplemented with 20% PI serum or 20% GPS. Statistical significance was assessed using one-way ANOVA with Tukey’s multiple comparisons test (95% CI, *P* < 0.0001), comparing all conditions to the PI control. All calculations were performed on GraphPad Prism 9.2.0 (GraphPad).

Bactericidal assays were conducted using serum from rats immunized with OspA, BAF, Chv2M, and BAF and Chv2M in combination. Near-complete (>95%) killing of *B. burgdorferi* B31-HE-OspC cells was observed with anti-BAF, anti-Chv2M, anti-BAF/Chv2M, and anti-OspA (control) antisera in the presence of GPS (exogenous complement source) (Fig. 4). High levels of cell killing (>60%) were also observed when the serum and cells were incubated with HI-GPS, demonstrating that both Ab-mediated complement-dependent and complement-independent mechanisms contribute to bactericidal activity.

**Fig 5:**
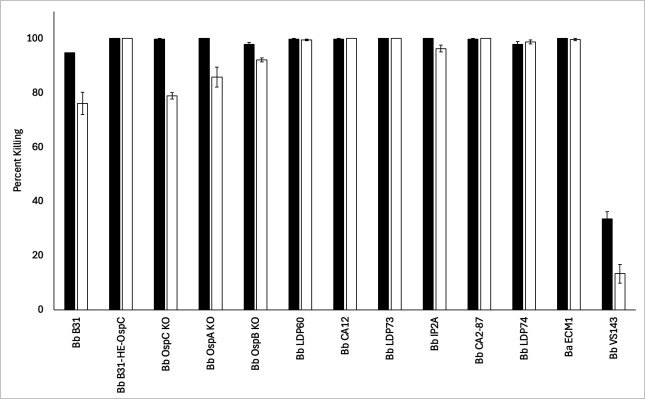
Anti-BAF/Chv2M serum kills *B. burgdorferi OspA, B, and C mutants and* strains of the Lyme disease spirochetes through Ab-mediated complement-dependent and complement-independent mechanisms. The ability of rat anti-BAF/Chv2M antiserum to kill *B. burgdorferi ospA, ospB*, or *ospC* gene deletion or gene interruption mutants (see Table 1) and several strains of Lyme disease spirochetes was measured. The test strains are indicated along the *x*-axis. Cells were incubated with anti-BAF/Chv2M antiserum with GPS (black bars) or HI-GPS (white bars). Visual counting by dark-field microscopy determined the number of live cells after treatment. Bb, *B. burgdorferi; Ba, B. afzelii*.

The bactericidal activity of the rat anti-BAF/Chv2M antiserum against *B. burgdorferi* strains and mutants was also assessed (Fig. 5). *OspA, ospB,* and *ospC* deletion mutants were included as test strains to determine if the absence of one of the vaccine target proteins attenuates Ab-mediated killing. Based on the complete killing of each mutant by vaccinal Abs, it can be concluded that killing is not dependent on the expression of any one particular Osp protein. Note that an *ftlA* deletion mutant was not generated and was unavailable for inclusion in these analyses. Vaccinal Abs also effectively killed multiple *B. burgdorferi* strains, indicating that the potential protective efficacy of the vaccine is not strain-dependent. The basis for the reduced killing of the European *B. burgdorferi* VS134 strain is unclear.

### Immunoglobulin isotyping using sera from mice immunized with BAF/Chv2M

Sera from immunized mice were screened for each IgG subclass using isotype-specific Abs (data not shown). IgG1 dominated, followed by IgG2b and IgG2a. The IgG3 response was minimal. The mouse isotype profiles reported here are consistent with earlier analyses of prototype OspC chimeritope antigens delivered in alum (41) and indicate a dominant Th2 response.

### Immunization with anti-BAF/Chv2M provides significant protection against infection with *B. burgdorferi*

**Fig 6:**
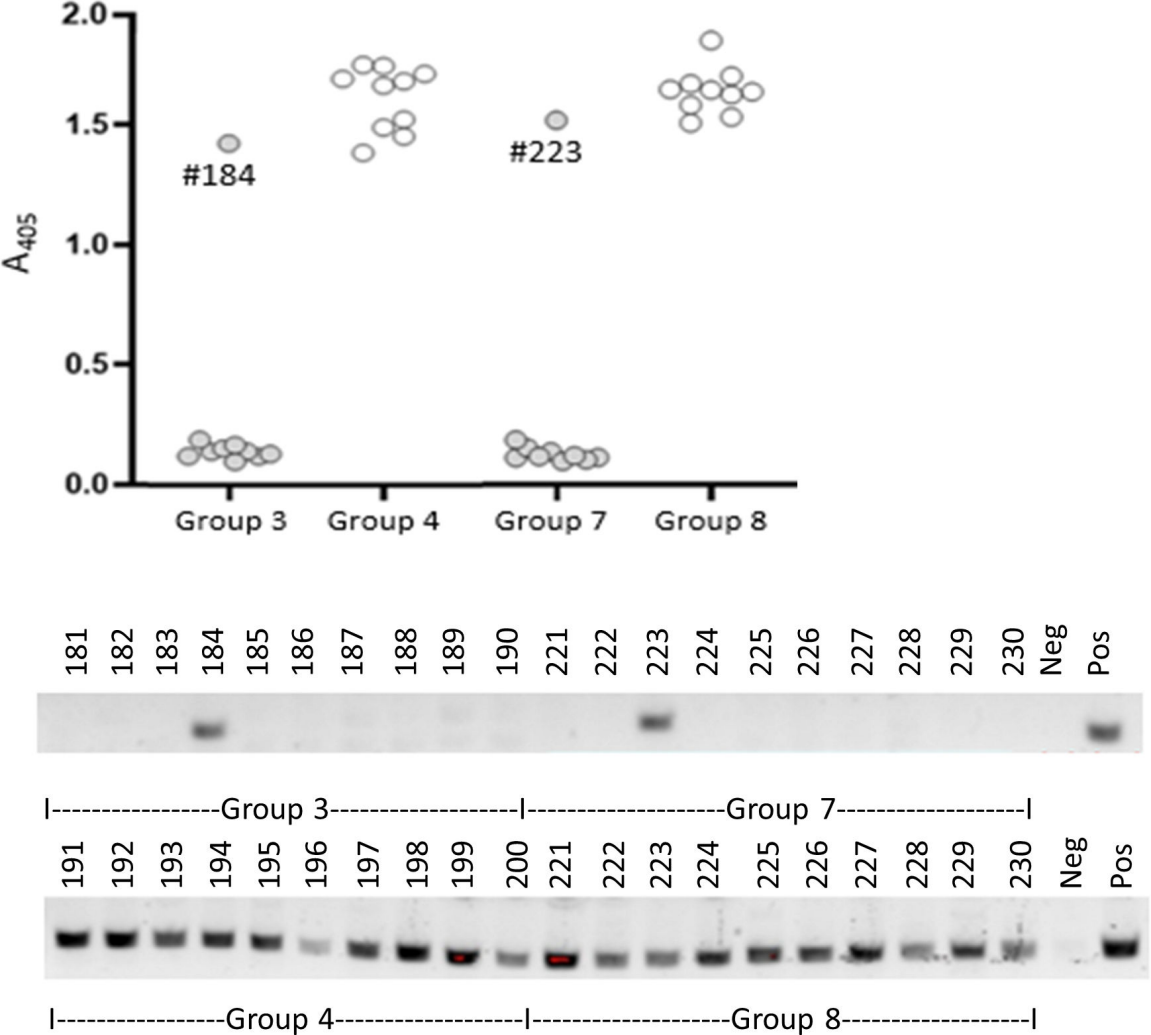
Immunization with BAF/Chv2M prevents infection with *B. burgdorferi*. Female (groups 3 and 4) and male (groups 7 and 8) mice were either immunized with BAF/Chv2M in Imject adjuvant (groups 3 and 5) or adjuvant alone (groups 4 and 8). Mouse groups are detailed in Table 2. The mice were challenged with 10^4^ *B. burgdorferi* B31 cells by subcutaneous needle inoculation. The mice were sacrificed 3 weeks post-challenge, and blood and ear tissue were collected. The top panel presents the results of ELISA analyses in which the serum (diluted 1:1,000) was tested for Abs to VlsE (full length; leader and his-tag removed). Each dot represents the average absorbance value of triplicate wells for each mouse. The animal identification numbers for immunized mice that were VlsE Ab-positive are indicated within the top panel. The lower panel presents the results of PCR (groups 3, 4, 5, and 8). DNA was extracted from ear tissue from each mouse and screened by PCR for *B. burgdorferi* DNA (*flaB* gene). The positive control reaction consisted of DNA extracted from ear tissue of a previously confirmed *B. burgdorferi* B31-infected mouse. The negative control reaction used DNA extracted from a non-infected mouse as template. The animal identification number for each mouse is indicated.

C3H/HeN mice were immunized, as detailed in Table 2. On day 42, mice in study groups 1 (10 females) and 5 (10 males) were euthanized, and the blood was harvested to assess Ab responses to immunization. All mice (*n* = 20) were Ab-positive for both immunogens (BAF and Chv2M). Based on this finding, the remaining mice (groups 2, 3, 6, and 7) were challenged and sacrificed 21 days later (day 63). Serum from all individual mice was screened by ELISA for Abs to VlsE, and DNA extracted from ear tissue was tested by PCR for the *B. burgdorferi flaB* gene (Fig. 6). Protection was scored as described in a recent OspA-based vaccine efficacy study (10). Mice were scored as protected if they were seronegative for Abs to VlsE and PCR-negative for *B. burgdorferi flaB*. The protective efficacy of a three-dose regimen of 25 µg of BAF and 25 µg Chv2M was 80% and 60% in female and male mice, respectively, and 70% overall. Increasing the amount of Chv2M to 50 µg in the third vaccine dose (groups 3 and 7) increased protective efficacy to 90% in both female and male mice (Table 2). Future analyses will assess the ability of the vaccine to protect against infection with representative isolates of each of the *Borreliella* species associated with disease in mammals.

## DISCUSSION

Here, we report on the development of a chimeric protein-based vaccine formulation for the prevention of Lyme disease that consists of two chimeric immunogens (BAF and Chv2M). The BAF protein, constructed using the *Borreliella* tick-phase OspB protein as the structural backbone, has two OspA_221-240_ ECD variants (#A1 and #A15) (22) incorporated into a surface-exposed loop domain (31). The OspB sequence immediately C-terminal to the insertion site of the OspA ECDs shares 82.4% aa identity over 16 residues with the OspA_221–240_ #A1 ECD including a perfect match over nine contiguous residues. The N-terminal domain of the FtlA protein (amino acid residues 19–143), which harbors one or more ECDs previously demonstrated to elicit bactericidal Abs (23), was added to the C-terminus to yield BAF. Chv2M, a chimeric epitope-based protein, comprises 20 different L5 and H5 ECDs from diverse OspC proteins (20, 24, 42, 43). We previously demonstrated that the L5 and H5 ECDs of OspC are largely responsible for the high specificity of Ab responses against diverse OspC proteins during natural infection and upon immunization with individual OspC proteins (44). The ECD composition of Chv2M was designed to elicit Abs that recognize diverse OspC types. While there is considerable diversity among OspC sequences (24, 41, 42, 45), within a phyletic cluster, amino acid sequence identity values are 95% or higher. At the inter-cluster level, amino acid identity values are as low as 60%. While immunization with a single OspC protein can protect against infection with *Borreliella* strains producing a closely related OspC protein (46), a multi-valent approach such as that employed here is essential to elicit broadly protective Abs.

ELISA analyses of serum harvested from mice or rats immunized with BAF/Chv2M demonstrated that the chimeric formulation is immunogenic. BAF elicited Abs that recognized all the individual proteins comprising the chimeric protein. In addition, anti-BAF Abs bound the major OspA variants and FtlB. The FtlA ECD of BAF shares 92% percent amino acid identity with the N-terminus of FtlB. Chv2M Abs bound all OspC proteins tested, including those associated with *B. mayonii* and *Borreliella* species from Europe and Asia.

The bactericidal activity of vaccinal Abs generated by immunization with BAF and Chv2M individually and BAF/Chv2M combined was measured. A caveat of assessing the bactericidal activity of Abs directed at OspC is that while OspC is expressed at high levels during early infection in mammals, its expression is low in cultivated LD spirochetes, and it is produced by only a small percentage of cells (44). To address this caveat and allow for an assessment of the bactericidal activity of anti-Chv2M Abs, *B. burgdorferi* B31-HE-OspC, which constitutively expresses OspC, was used as the test strain. Rat anti-OspA, anti-Chv2M, and anti-BAF/Chv2M Abs effectively killed all cells via an Ab-mediated complement-dependent mechanism. Significant killing (~55%–75%) was also observed when Abs were incubated with cells in the presence of HI-GPS, indicating Ab-mediated complement-independent killing. Previous studies have postulated that two anti-OspB IgG class monoclonal Abs (H6831 and CB2) kill cells through a complement-independent mechanism, presumably osmolysis due to membrane disruption (47–49). While we have not directly assessed the mechanistic basis of Ab-mediated complement-independent killing by anti-BAF/Chv2M, membrane disruption, as suggested by Benach and colleagues, is likely a contributing factor. Interestingly, the binding site of H6831 has been localized to the region in and around residue 253 of OspB. This is the same region into which the OspA ECDs were incorporated into OspB. Complement-independent killing could be an important contributor to killing *B. burgdorferi* in ticks, as tick salivary proteins have been demonstrated to inhibit complement activity (50). The mechanism associated with complement-independent killing remains to be determined.

The bactericidal activity of Abs from mice immunized with BAF/Chv2M against eight different LD spirochete strains was also assessed. Near-complete killing via Ab-mediated complement-dependent and complement-independent mechanisms was observed for all strains except *B. burgdorferi* VS134 (Table 1). The basis for this strain’s lower level of killing is unknown. *B. burgdorferi* mutant strains that do not produce OspA (*B. burgdorferi* 2E6) (27) or OspC (*B. burgdorferi* B31 clone ∆*ospC*) were also killed by anti-BAF/Chv2M Abs. This result demonstrates that the bactericidal activity is mediated by Abs directed at multiple protein targets. The ability of the chimerics to act through a complement-dependent mechanism is consistent with earlier studies focused on prototype OspC chimeritopes (42, 45).

Immunization with BAF/Chv2M provided significant protection against challenge with *B. burgdorferi* B31 delivered by needle inoculation. Of the 40 mice challenged with three vaccine doses, 80% were protected. This increased to 90% for mice that received an increased amount of Chv2M in the final vaccine dose. Protection was determined based on the lack of seroconversion to VlsE, a common diagnostic marker, and the absence of detectable *B. burgdorferi flaB* DNA by PCR. All vehicle control mice, one immunized male and one immunized female, seroconverted to VlsE and were *flaB* PCR-positive and, thus, scored as infected.

This study establishes proof of principle for a chimeric immunogen vaccine formulation that elicits Abs to multiple targets on the *B. burgdorferi* cell surface produced during the tick and mammalian stages of the enzootic cycle. The development of a multivalent vaccine formulation represents a significant step forward in developing a next-generation multivalent vaccine to prevent Lyme disease.

## References

[1] Mead P. 2022. Epidemiology of Lyme disease. Infect Dis Clin North Am 36:495–521. doi:10.1016/j.idc.2022.03.004 36116831

[2] Vandekerckhove O, De Buck E, Van Wijngaerden E. 2021. Lyme disease in Western Europe: an emerging problem? A systematic review. Acta Clin Belg 76:244–252. doi:10.1080/17843286.2019.1694293 31739768

[3] Estrada-Peña A, Cutler S, Potkonjak A, Vassier-Tussaut M, Van Bortel W, Zeller H, Fernández-Ruiz N, Mihalca AD. 2018. An updated meta-analysis of the distribution and prevalence of Borrelia burgdorferi s.l. in ticks in Europe. Int J Health Geogr 17:41. doi:10.1186/s12942-018-0163-7 30514310 PMC6319795

[4] Gingerich MC, Nair N, Azevedo JF, Samanta K, Kundu S, He B, Gomes-Solecki M. 2024. Intranasal vaccine for Lyme disease provides protection against tick transmitted Borrelia burgdorferi beyond one year. NPJ Vaccines 9:33. doi:10.1038/s41541-023-00802-y 38360853 PMC10869809

[5] Nayak A, Schüler W, Seidel S, Gomez I, Meinke A, Comstedt P, Lundberg U. 2020. Broadly protective multivalent OspA vaccine against lyme borreliosis, developed based on surface shaping of the C-terminal fragment. Infect Immun 88:e00917-19. doi:10.1128/IAI.00917-19 31932330 PMC7093141

[6] Fikrig E, Barthold SW, Kantor FS, Flavell RA. 1990. Protection of mice against the Lyme disease agent by immunizing with recombinant OspA. Science 250:553–556. doi:10.1126/science.2237407 2237407

[7] Parenti D. 1999. Lyme disease vaccine--LYMErix. Conn Med 63:570. 10576966

[8] Comstedt P, Hanner M, Schüler W, Meinke A, Lundberg U. 2014. Design and development of a novel vaccine for protection against Lyme borreliosis. PLoS One 9:e113294. doi:10.1371/journal.pone.0113294 25409015 PMC4237411

[9] Comstedt P, Hanner M, Schüler W, Meinke A, Schlegl R, Lundberg U. 2015. Characterization and optimization of a novel vaccine for protection against Lyme borreliosis. Vaccine (Auckl) 33:5982–5988. doi:10.1016/j.vaccine.2015.07.095 26277070

[10] Comstedt P, Schüler W, Meinke A, Lundberg U. 2017. The novel Lyme borreliosis vaccine VLA15 shows broad protection against Borrelia species expressing six different OspA serotypes. PLoS ONE 12:e0184357. doi:10.1371/journal.pone.0184357 28863166 PMC5581183

[11] O’Bier NS, Hatke AL, Camire AC, Marconi RT. 2021. Human and veterinary vaccines for Lyme disease. Curr Issues Mol Biol 42:191–222. doi:10.21775/cimb.042.191 33289681 PMC7946718

[12] Bhowmik D, Bhuyan A, Gunalan S, Kothandan G, Kumar D. 2023. In silico and immunoinformatics based multiepitope subunit vaccine design for protection against visceral leishmaniasis. J Biomol Struct Dyn:1–22. doi:10.1080/07391102.2023.2252901 37655736

[13] Jalalvand A, Fotouhi F, Bahramali G, Bambai B, Farahmand B. 2023. In silico design of a trivalent multi-epitope global-coverage vaccine-candidate protein against influenza viruses: evaluation by molecular dynamics and immune system simulation. J Biomol Struct Dyn:1–17. doi:10.1080/07391102.2023.2292293 38088331

[14] Moqbel Hassan Alzubaydi N, Oun Ali Z, Al-Asadi S, Al-Kahachi R. 2024. Design and characterization of a multi-epitope vaccine targeting Chlamydia abortus using immunoinformatics approach. J Biomol Struct Dyn 42:6660–6677. doi:10.1080/07391102.2023.2240891 37774751

[15] Nayak AK, Chakraborty A, Shukla S, Kumar N, Samanta S. 2024. An immunoinformatic approach for developing a multi-epitope subunit vaccine against Monkeypox virus. In Silico Pharmacol 12:42. doi:10.1007/s40203-024-00220-5 38746047 PMC11089034

[16] Pal A, Pyne N, Paul S. 2023. In-silico designing of a multi-epitope vaccine against SARS-CoV2 and studying the interaction of the vaccine with Alpha, Beta, Delta and Omicron variants of concern. Curr Drug Discov Technol 20:e090922208713. doi:10.2174/1570163819666220909114900 36093818

[17] Rashidi S, Faraji SN, Mamaghani AJ, Hatam S, Kazemi B, Bemani P, Tabaei SJS, Hatam G. 2022. Bioinformatics analysis for the purpose of designing a novel multi-epitope DNA vaccine against Leishmania major. Sci Rep 12:18119. doi:10.1038/s41598-022-22646-7 36302830 PMC9612607

[18] Tirziu A, Paunescu V. 2022. Cytotoxic T-cell-based vaccine against SARS-CoV-2: a hybrid immunoinformatic approach. Vaccines (Basel) 10:218. doi:10.3390/vaccines10020218 35214676 PMC8878688

[19] Marconi RT, Garcia-Tapia D, Hoevers J, Honsberger N, King VL, Ritter D, Schwahn DJ, Swearingin L, Weber A, Winkler MTC, Millership J. 2020. VANGUARDcrLyme: a next generation Lyme disease vaccine that prevents B. burgdorferi infection in dogs. Vaccine: X 6:100079. doi:10.1016/j.jvacx.2020.100079 33336185 PMC7733144

[20] Earnhart CG, Buckles EL, Dumler JS, Marconi RT. 2005. Demonstration of OspC type diversity in invasive human lyme disease isolates and identification of previously uncharacterized epitopes that define the specificity of the OspC murine antibody response. Infect Immun 73:7869–7877. doi:10.1128/IAI.73.12.7869-7877.2005 16299277 PMC1307023

[21] Marconi RT, Honsberger N, Teresa Winkler M, Sobell N, King VL, Wappel S, Hoevers J, Xu Z, Millership J. 2020. Field safety study of VANGUARDcrLyme: a vaccine for the prevention of Lyme disease in dogs. Vaccine: X 6:100080. doi:10.1016/j.jvacx.2020.100080 33336186 PMC7733143

[22] Izac JR, Oliver LD, Earnhart CG, Marconi RT. 2017. Identification of a defined linear epitope in the OspA protein of the Lyme disease spirochetes that elicits bactericidal antibody responses: implications for vaccine development. Vaccine (Auckl) 35:3178–3185. doi:10.1016/j.vaccine.2017.04.079 PMC8203411 28479174

[23] Camire AC, O’Bier NS, Patel DT, Cramer NA, Straubinger RK, Breitschwerdt EB, Funk RA, Marconi RT. 2022. FtlA and FtlB are candidates for inclusion in a next-generation multiantigen subunit vaccine for Lyme disease. Infect Immun 90:e0036422. doi:10.1128/iai.00364-22 36102656 PMC9584329

[24] Buckles EL, Earnhart CG, Marconi RT. 2006. Analysis of antibody response in humans to the type A OspC loop 5 domain and assessment of the potential utility of the loop 5 epitope in Lyme disease vaccine development. Clin Vaccine Immunol 13:1162–1165. doi:10.1128/CVI.00099-06 17028218 PMC1595320

[25] Barbour AG, Burgdorfer W, Hayes SF, Péter O, Aeschlimann A. 1983. Isolation of a cultivable spirochete fromIxodes ricinus ticks of Switzerland. Curr Microbiol 8:123–126. doi:10.1007/BF01566969

[26] Earnhart CG, Leblanc DV, Alix KE, Desrosiers DC, Radolf JD, Marconi RT. 2010. Identification of residues within ligand-binding domain 1 (LBD1) of the Borrelia burgdorferi OspC protein required for function in the mammalian environment. Mol Microbiol 76:393–408. doi:10.1111/j.1365-2958.2010.07103.x 20199597 PMC2917209

[27] Battisti JM, Bono JL, Rosa PA, Schrumpf ME, Schwan TG, Policastro PF. 2008. Outer surface protein A protects Lyme disease spirochetes from acquired host immunity in the tick vector. Infect Immun 76:5228–5237. doi:10.1128/IAI.00410-08 18779341 PMC2573341

[28] Tilly K, Casjens S, Stevenson B, Bono JL, Samuels DS, Hogan D, Rosa P. 1997. The Borrelia burgdorferi circular plasmid cp26: conservation of plasmid structure and targeted inactivation of the ospC gene. Mol Microbiol 25:361–373. doi:10.1046/j.1365-2958.1997.4711838.x 9282748

[29] Lane RS, Pascocello JA. 1989. Antigenic characteristics of Borrelia burgdorferi isolates from ixodid ticks in California. J Clin Microbiol 27:2344–2349. doi:10.1128/jcm.27.10.2344-2349.1989 2685030 PMC267021

[30] Baranton G, Postic D, Saint Girons I, Boerlin P, Piffaretti JC, Assous M, Grimont PA. 1992. Delineation of Borrelia burgdorferi sensu stricto, Borrelia garinii sp. nov., and group VS461 associated with Lyme borreliosis. Int J Syst Bacteriol 42:378–383. doi:10.1099/00207713-42-3-378 1380285

[31] Becker M, Bunikis J, Lade BD, Dunn JJ, Barbour AG, Lawson CL. 2005. Structural investigation of Borrelia burgdorferi OspB, a bactericidal Fab target. J Biol Chem 280:17363–17370. doi:10.1074/jbc.M412842200 15713683

[32] Schuler EJA, Patel DT, Marconi RT. 2023. The leptospiral OmpA-like protein (Loa22) is a surface-exposed antigen that elicits bactericidal antibody against heterologous Leptospira. Vacc X 15:100382. doi:10.1016/j.jvacx.2023.100382 PMC10506094 37727366

[33] Wilkins MR, Gasteiger E, Bairoch A, Sanchez JC, Williams KL, Appel RD, Hochstrasser DF. 1999. Protein identification and analysis tools in the ExPASy server. Methods Mol Biol 112:531–552. doi:10.1385/1-59259-584-7:531 10027275

[34] Jumper J, Evans R, Pritzel A, Green T, Figurnov M, Ronneberger O, Tunyasuvunakool K, Bates R, Žídek A, Potapenko A, et al.. 2021. Highly accurate protein structure prediction with AlphaFold. Nature New Biol 596:583–589. doi:10.1038/s41586-021-03819-2 PMC8371605 34265844

[35] Meng EC, Goddard TD, Pettersen EF, Couch GS, Pearson ZJ, Morris JH, Ferrin TE. 2023. UCSF ChimeraX: tools for structure building and analysis. Protein Sci 32:e4792. doi:10.1002/pro.4792 37774136 PMC10588335

[36] Izac JR, O’Bier NS, Oliver LD, Camire AC, Earnhart CG, LeBlanc Rhodes DV, Young BF, Parnham SR, Davies C, Marconi RT. 2020. Development and optimization of OspC chimeritope vaccinogens for Lyme disease. Vaccine (Auckl) 38:1915–1924. doi:10.1016/j.vaccine.2020.01.027 PMC7085410 31959423

[37] Patel DT, O’Bier NS, Schuler EJA, Marconi RT. 2021. The Treponema denticola DgcA protein (TDE0125) is a functional diguanylate cyclase . Pathog Dis 79. doi:10.1093/femspd/ftab004 33452878

[38] Cramer NA, Socarras KM, Earl J, Ehrlich GD, Marconi RT. 2024. Borreliella burgdorferi factor H-binding proteins are not required for serum resistance and infection in mammals. Infect Immun 92:e0052923. doi:10.1128/iai.00529-23 38289123 PMC10929407

[39] Johnson TL, Graham CB, Hojgaard A, Breuner NE, Maes SE, Boegler KA, Replogle AJ, Kingry LC, Petersen JM, Eisen L, Eisen RJ. 2017. Isolation of the Lyme disease spirochete Borrelia mayonii from naturally infected rodents in Minnesota. J Med Entomol 54:1088–1092. doi:10.1093/jme/tjx062 28444198 PMC5664935

[40] Pritt BS, Respicio-Kingry LB, Sloan LM, Schriefer ME, Replogle AJ, Bjork J, Liu G, Kingry LC, Mead PS, Neitzel DF, Schiffman E, Hoang Johnson DK, Davis JP, Paskewitz SM, Boxrud D, Deedon A, Lee X, Miller TK, Feist MA, Steward CR, Theel ES, Patel R, Irish CL, Petersen JM. 2016. Borrelia mayonii sp. nov., a member of the Borrelia burgdorferi sensu lato complex, detected in patients and ticks in the upper midwestern United States. Int J Syst Evol Microbiol 66:4878–4880. doi:10.1099/ijsem.0.001445 27558626 PMC5214957

[41] Earnhart CG, Marconi RT. 2007. Construction and analysis of variants of a polyvalent Lyme disease vaccine: approaches for improving the immune response to chimeric vaccinogens. Vaccine (Auckl) 25:3419–3427. doi:10.1016/j.vaccine.2006.12.051 PMC2696934 17239505

[42] Earnhart CG, Buckles EL, Marconi RT. 2007. Development of an OspC-based tetravalent, recombinant, chimeric vaccinogen that elicits bactericidal antibody against diverse Lyme disease spirochete strains. Vaccine (Auckl) 25:466–480. doi:10.1016/j.vaccine.2006.07.052 16996663

[43] Earnhart CG, Marconi RT. 2007. OspC phylogenetic analyses support the feasibility of a broadly protective polyvalent chimeric Lyme disease vaccine. Clin Vaccine Immunol 14:628–634. doi:10.1128/CVI.00409-06 17360854 PMC1865620

[44] Oliver LD, Earnhart CG, Virginia-Rhodes D, Theisen M, Marconi RT. 2016. Antibody profiling of canine IgG responses to the OspC protein of the Lyme disease spirochetes supports a multivalent approach in vaccine and diagnostic assay development. The Vet J 218:27–33. doi:10.1016/j.tvjl.2016.11.001 27938705

[45] Earnhart CG, Marconi RT. 2007. An octavalent lyme disease vaccine induces antibodies that recognize all incorporated OspC type-specific sequences. Hum Vaccin 3:281–289. doi:10.4161/hv.4661 17921702

[46] Bockenstedt LK, Hodzic E, Feng S, Bourrel KW, de Silva A, Montgomery RR, Fikrig E, Radolf JD, Barthold SW. 1997. Borrelia burgdorferi strain-specific OspC-mediated immunity in mice. Infect Immun 65:4661–4667. doi:10.1128/iai.65.11.4661-4667.1997 9353047 PMC175668

[47] Coleman JL, Rogers RC, Benach JL. 1992. Selection of an escape variant of Borrelia burgdorferi by use of bactericidal monoclonal antibodies to OspB. Infect Immun 60:3098–3104. doi:10.1128/iai.60.8.3098-3104.1992 1639477 PMC257287

[48] Escudero R, Halluska ML, Backenson PB, Coleman JL, Benach JL. 1997. Characterization of the physiological requirements for the bactericidal effects of a monoclonal antibody to OspB of Borrelia burgdorferi by confocal microscopy. Infect Immun 65:1908–1915. doi:10.1128/iai.65.5.1908-1915.1997 9125579 PMC175240

[49] Sadziene A, Jonsson M, Bergström S, Bright RK, Kennedy RC, Barbour AG. 1994. A bactericidal antibody to Borrelia burgdorferi is directed against a variable region of the OspB protein. Infect Immun 62:2037–2045. doi:10.1128/iai.62.5.2037-2045.1994 7513309 PMC186463

[50] Denisov SS, Ippel JH, Castoldi E, Mans BJ, Hackeng TM, Dijkgraaf I. 2021. Molecular basis of anticoagulant and anticomplement activity of the tick salivary protein Salp14 and its homologs. J Biol Chem 297:100865. doi:10.1016/j.jbc.2021.100865 34118237 PMC8294578

